# Investigating the applicability of novel hydrate dissociation inhibitors in oilwell cement through molecular simulations

**DOI:** 10.1038/s41598-024-65935-z

**Published:** 2024-06-28

**Authors:** Yuhuan Bu, Zilong Lu, Chang Lu, Huajie Liu, Shenglai Guo, Hexing Liu

**Affiliations:** 1 School of Petroleum Engineering, China University of Petroleum (East China), Qingdao, Shandong China; 2 Key Laboratory of Oilfield Chemistry of Shandong Province, Key Laboratory of Unconventional Oil and Gas Development of Ministry of Education, China University of Petroleum (East China), Qingdao, 266580 Shandong China; 3 School of Environmental and Municipal Engineering, Qingdao University of Technology, Qingdao, Shandong China; 4 China National Offshore Oil Corporation (China), Zhan Jiang, China

**Keywords:** CSH model of oilwell cement, Hydrate formation cementing additive, Molecular dynamics simulation, Hydrate dissociation inhibitor, Energy, Chemical engineering, Civil engineering, Energy harvesting, Renewable energy, Chemical physics

## Abstract

In the field of hydrate formation cementing, the method of developing the low hydration exothermic cement systems cannot effectively solve the problem of hydrate dissociation caused by the hydration heat release of cement. Therefore, we proposed a new approach to address this issue by employing cement additives that can effectively delay the dissociation of hydrate. In our previous work, we designed a novel hydrate dissociation inhibitor, PVCap/dmapma, however, its applicability with cement slurry remains unverified. In this study, we established a more realistic model of oilwell cement gel based on experimental data. Additionally, we investigated the potential effects of PVCap/dmapma on the microstructure and mechanical properties of cement gel through molecular simulations. The results suggest that PVCap/dmapma has no negative effect on the performance of cement slurry compared to Lecithin. By adding PVCap/dmapma to cement slurry, the problem of cementing in hydrate formations is expected to be solved.

## Introduction

For offshore oil and gas drilling and completion operations, hydrate formation is easily formed in shallow formations of deep water^1^. During the deep-water drilling process, the hydrate formation could be drilled, and the substantial heat generated by the bit during rock cutting and fracturing can trigger hydrate dissociation, potentially leading to major accidents, including formation collapse^2^. To solve this problem, BP added a certain amount of Lecithin to drilling fluid and successfully stabilized the hydrate formation in the permafrost zone at the southeast edge of the Alaska Arctic region^3,4^. Lecithin is the only chemical applied in the oil and gas industry that has successfully inhibited hydrate dissociation currently.

In the cementing operation in shallow formations of deep water, the hydration heat of cement will cause the dissociation of hydrate around the well wall during waiting on cement time after the injection of cement slurry. In the ordinary silicate low-hydration exothermic cement slurry system, the peak temperature of the hydration process typically approximates 60 °C, which is much higher than the phase equilibrium temperature of hydrate in shallow formations of deep water. On one hand, the cementation interface between the cement sheath and the casing will be damaged and a micro-gap will be generated, which will lead to the failure of cement sealing integrity and even blowout. On the other hand, the cement strength cannot develop well due to the appearance of CH_4_ gas in the slurry, which cannot meet the quality requirements of deepwater cementing^5–7^. Furthermore, the interaction mechanism between hydrates and cement slurry remains unrevealed, although some literature that sheds light on the influence mechanism of clay minerals on hydrate provides good reference significance^8,9^.

To solve this problem, scholars have developed a variety of low-hydration exothermic cement slurry systems^10–14^. However, whether low-hydration heat materials or phase-change energy storage microspheres is added to reduce the hydration heat, the peak value of the minimum exothermic temperature of cement slurry systems still reaches 38 °C under the open environment which still will lead to the dissociation of hydrate. Therefore, how to inhibit the dissociation of hydrate is an urgent problem to be solved in the field of deepwater cementing.

We try to delay the dissociation of hydrate by adding additives to cement slurry while reducing the hydration heat of cement slurry. To explore whether the hydrate dissociation inhibitor (Lecithin) for drilling fluid applies to cement slurry, Bu et al.^15^ studied the effect of Lecithin on the thickening and mechanical properties of cement slurry. The result demonstrated that phosphate groups will chelate with calcium ions, resulting in the abnormality of cement slurry thickening. The compressive strength and elastic modulus of cement containing 0.5–1.5 wt% Lecithin decreased significantly. They believe that Lecithin will lead to the decalcification of Calcium-Silicate-Hydrate (CSH) gel, which will affect its mechanical properties. Thus, Lecithin is not suitable for the field of hydrate formation cementing. A hydrate dissociation inhibitor suitable for cementing slurry systems needs to be developed.

In our previous work^16^, we designed a new hydrate dissociation inhibitor (PVCap/dmapma) based on the hydrate stability characteristics and the removal of the functional groups that affect the mechanical properties of cement. PVCap/dmapma can adsorb on the surface of hydrate, forming a network structure that hinders methane gas escape and ultimately achieves the effect of inhibiting hydrate dissociation. However, the applicability of PVCap/dmapma in cement slurry system remains to be validated.

In our research, we have conducted a comparative analysis at the nanoscale, utilizing molecular simulations, to investigate the effects of Lecithin and PVCap/dmapma as cement additives on the properties of CSH gel. Molecular dynamics (MD) simulation serves as a crucial technology for comprehending the atomic-level physical and chemical properties of cement gels, which has a resolution ratio that remains inaccessible to traditional experimental and numerical simulation techniques. Over several decades, molecular simulation technology has garnered widespread adoption among scholars for simulating inorganic minerals and CSH gel^17–19^, which can accurately calculate the mechanical and thermodynamic properties of CSH^20–22^.

PVCap/dmapma should meet the following requirements: avoid containing functional groups similar to phosphate groups, not chelating with calcium ions, and not leading to the decalcification of CSH gel. According to the previous studies^23–25^, polymer molecules can exist in two states in cement slurry from the perspective of molecular simulation: ① movement and migration in the capillary pores of cement slurry; ② intercalation in cement particles or adsorption at the defects. Therefore, we respectively studied the "movement and migration" and "internal intercalation or adsorption" of additives in cement slurry, and comprehensively evaluated the impact of PVCap/dmapma on the performance of cement slurry: ① By studying the movement and adsorption state of PVCap/dmapma and Lecithin in capillary pores of CSH gel, the impact of PVCap/dmapma on the microstructure of CSH gel was compared and analyzed; ② Further Exploring the reason why Lecithin is not suitable for cement slurry; ③ The impact of PVCap/dmapma intercalation in CSH gel on the microstructure and mechanical properties of CSH gel.

## Models and simulation methods

### Grand canonical Monte Carlo simulation

Grand Canonical Monte Carlo (GCMC) simulation is usually used to calculate the adsorption capacity of small molecular gas or liquid in solid materials. During the simulation, the volume, temperature, and chemical potential of the system should be fixed (16 °C, 0 eV)^26^. The water molecules from the liquid source would be supplied to CSH skeleton until the system tends to saturation. Temperature and chemical potential determine the molecular number of the bound water. The accuracy of the bound water content in CSH model can be verified by the thermogravimetric analysis^27–29^.

### CSH gel model of low-temperature oilwell cement

**Figure 1:**
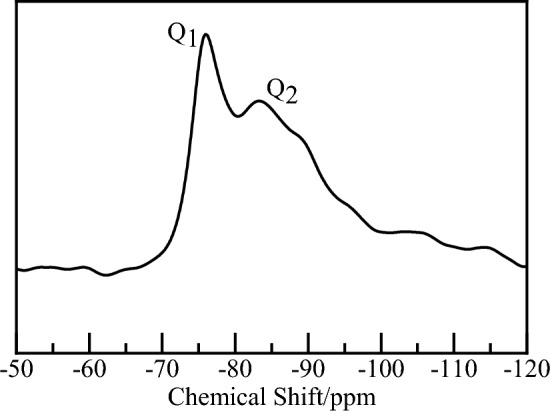
^29^Si-NMR spectrum of oilwell cement’s hydration product.

Oilwell cement produced by Sichuan Jiahua Enterprise was selected as the raw material, and the cement slurry was cured at a temperature of 16 °C for 7 days (the water-cement ratio is 0.5). Then the ^29^Si-NMR spectrum (as shown in Fig. 1) was obtained by solid-state nuclear magnetic resonance, and the polymerization state information of the silicon-oxygen tetrahedron was calculated. The Ca/Si ratio in CSH gel was determined to be about 1.50 by the low-hydration exothermic cement formula. The content of bound water in cement was calculated by thermogravimetric analysis.

At present, the ^29^Si-NMR spectrum is widely used to determine the polymerization state information of silicon-oxygen tetrahedrons in CSH gel, which is of great significance to the study of CSH gel structure^30–32^. Scholars usually use Q_n_ distribution to characterize the connection degree of silicon chains: Q_0_ represents silicon-oxygen tetrahedral monomer; Q_1_ represents the silicon atom connected with a bridging oxygen atom; Q_2_ represents the silicon atom bridging two oxygen atoms.

**Figure 2:**
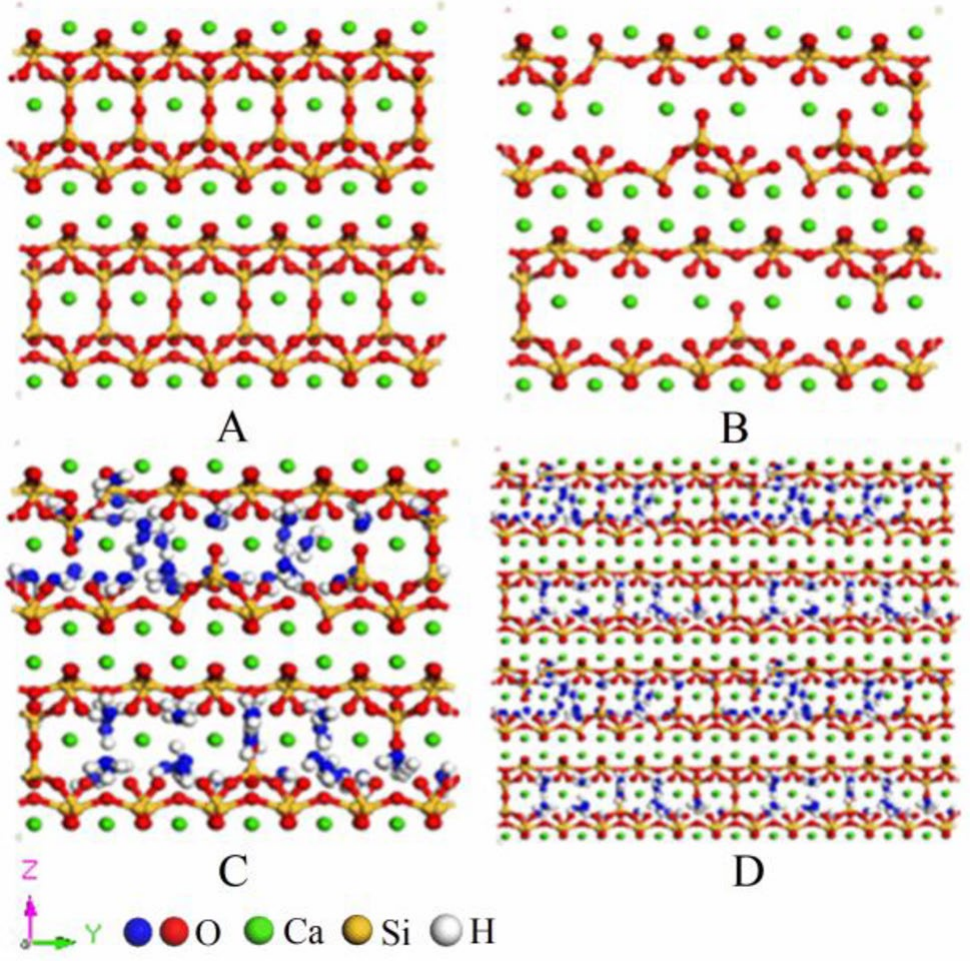
Establishment process of CSH gel model: (**A**) The CSH skeleton after removing water molecules, (**B**) The CSH skeleton after breaking silicon chains, (**C**) GCMC simulation and (**D**) CSH gel model after crystal cell expansion.

Through calculating the respective areas of the two absorption peaks Q_1_ and Q_2_, the distribution of silicon chain polymerization in the hydration products of low-temperature oilwell cement was obtained: Q_1_: Q_2_ = 55.87%: 44.13%. Then, the Tobermorite11Å model by Hamid^33^ was used as the basement, and the water molecules in the model were removed, leaving only the CSH skeleton (as shown in Fig. 2A). The complete silicon chain was randomly broken according to the Q_n_ distribution. Add or delete calcium ions in the model according to Ca/Si ratio (as shown in Fig. 2B). Then conduct GCMC simulation for the CSH skeleton until the model is saturated with water molecules (as shown in Fig. 2C). Finally, carrying out the crystal cell expansion of 2 × 2 × 2 size, the length, width, and height of the CSH gel model is 4.4635 nm, 4.434 nm, and 4.554 nm respectively (as shown in Fig. 2D). In the CSH gel model, red is oxygen atom, green is calcium atom, yellow is silicon atom, and white is hydrogen atom. To highlight the adsorbed water molecules, blue is used here to indicate the oxygen atoms in the bound water.

**Figure 3:**
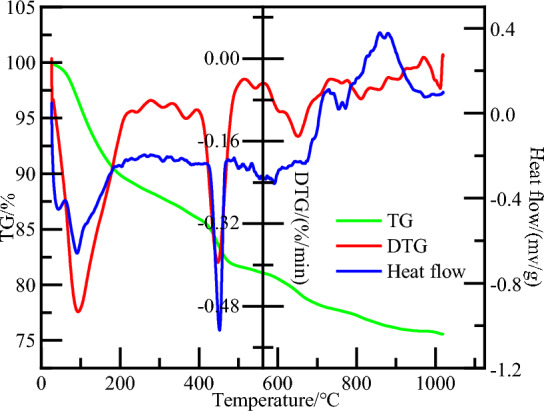
Weight loss process of CSH gel.

TG curve, DTG curve, and Heat flow curve of hydration product of oilwell cement were obtained through Thermogravimetric analysis test (as shown in Fig. 3). According to the TG curve, the bound water content was calculated at 10.53%. The bound water content of the model after GCMC simulation is 9.57%, which is in line with the experimental data generally, so it also verifies the accuracy of the CSH gel model.

Although we have modified Tobermorite11Å model according to the ^29^Si-NMR spectrum, it still does not reach the complexity of the real-world. Furthermore, the classical molecular dynamics model cannot focus on the dynamic change process of its structural components. The establishment of CSH gel model based on experimental data possesses an advantage over the direct utilization of perfect jennite or tobermorite mineral crystals as surrogates. This model incorporates a higher degree of silicon chain defects, structural complexity, and disorder, thereby rendering it a more realistic model.

**Figure 4:**
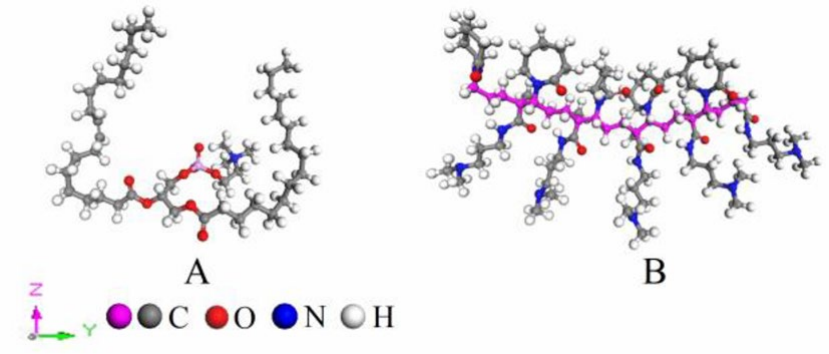
The 3D structure of molecules: (**A**) Lecithin and (**B**) PVCap/dmapma.

The chemical structural formula of Lecithin is C_42_H_84_O_9_PN; The new hydrate dissociation inhibitor (PVCap/dmapma) is formed by the polymerization of dmapma (C_9_H_18_N_2_O) and N-vcap (C_8_H_13_NO). The 3D structure of the two molecules is shown in Fig. 4.

### CSH gel composite models

**Figure 5:**
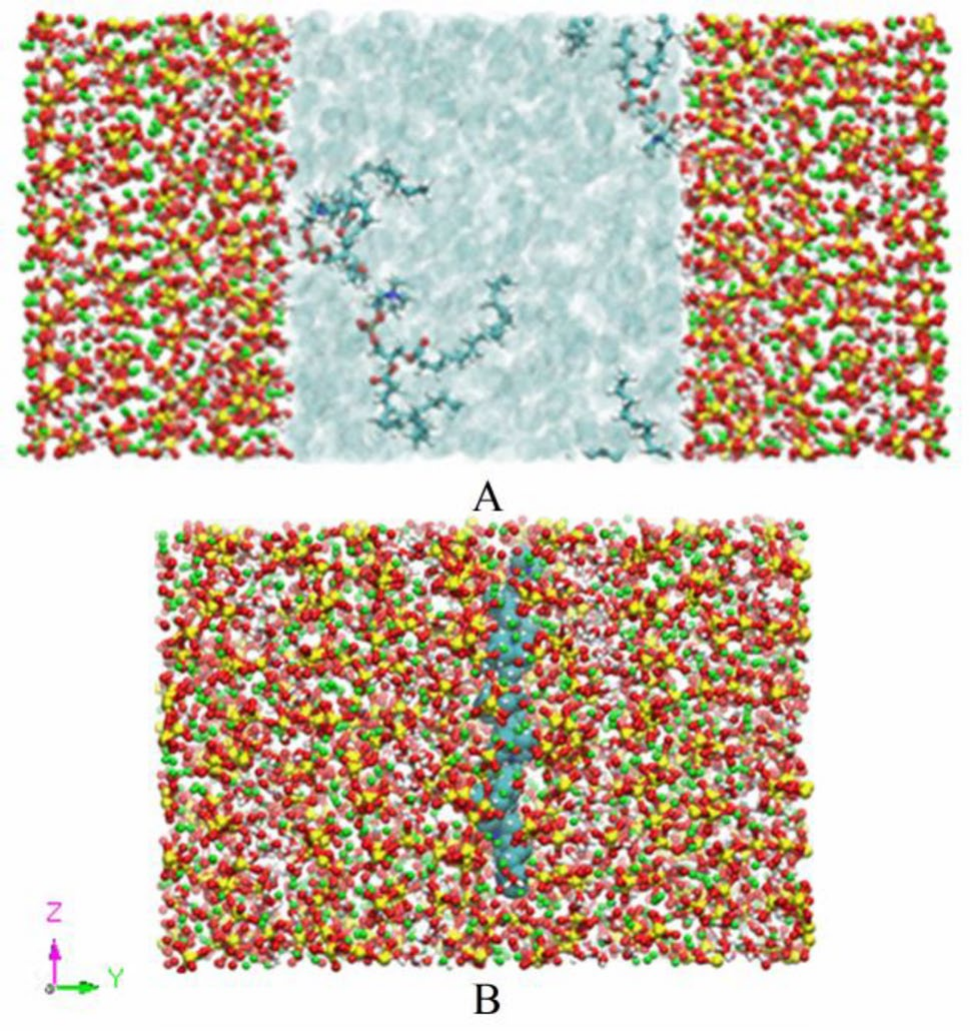
CSH composite model: (**A**) Capillary pore of CSH gel model and (**B**) Intercalation of PVCap/dmapma in CSH gel Model.

To study the two distribution states of additives in CSH gel, the two composite models were established: ① CSH gel capillary pore model (as shown in Fig. 5A): the upper and lower layers are CSH gel, and the middle layer is the capillary pore of 4.0 nm wide. The density of water in the capillary pore is 1.0 g/cm^3^, which conforms to the actual situation. ② The additive was intercalated into the CSH gel model (as shown in Fig. 5B): PVCap/dmapma is coupled between the silicon chain layers of the two-layer CSH gel. In the CSH gel model, red is oxygen atom, green is calcium atom, yellow is silicon atom, and white is hydrogen atom. The light blue area in the capillary pore is water molecules, cyan is carbon atom, dark yellow is phosphorus atom, and blue is nitrogen atom.

### Simulation methods

CSH gel is described by the ClayFF force field^34,35^, the CvFF force field^36^ is used for Lecithin and PVCap/dmapma, and water molecules use the SPC model^37^. The Jorgensen mixing rule is adopted for the coupling between different force fields^38^. In the study of the interfacial properties of polymer molecules and inorganic cement materials, many scholars have verified the accuracy of the combination of the ClayFF and CvFF force field^39–41^.

Because of the coupling between solid and liquid phases in model ①, it is necessary to fix the CSH gel first, and the Steepest Descent method^42^ and Conjugate method^43^ are used for energy minimization. Then, under the NVT ensemble (constant atom number, constant volume, constant temperature), the full relaxation was conducted to the system for 500 ps (timesteps is 0.5 fs). Finally, the fixation was removed, and the molecular dynamics simulation was carried out for 2000 ps under the NPT ensemble (constant atom number, constant pressure, constant temperature) with a temperature of 16 °C and pressure of 0.1 MPa (timesteps is 1.0 fs). The simulation parameters are set based on the actual conditions encountered in deepwater cementing operations.

For model ②, the Steepest Descent method and Conjugate method are directly used to minimize energy for the system. Then the pre-relaxation of 200 ps was carried out under the NVT ensemble to eliminate the internal stress of system. Then molecular dynamics simulation was carried out for 500 ps under the NPT ensemble with a temperature of 16 °C and pressure of 0.1 MPa so that PVCap/dmapma could fully interact with the constituent atoms of CSH gel. Finally, the “deform” command was used to stretch or compress the entire system.

In the LAMMPS package, the temperature and pressure are controlled by the Nose–Hoover thermostat^44,45^ and Berendsen barostat^46^ respectively. The three-dimensional boundary is treated by periodic boundary conditions. The cutoff radius of L–J potential for short-range interaction is 1.25 nm, and the Ewald algorithm is used for long-range electrostatic interaction.

## Result and discussion

### Interface interaction between monomer molecules and CSH gel

In CSH-polymer system, the polarity of functional groups and the diffusion movement characteristics of polymer molecules determine the affinity between molecules and CSH gel. The stronger the interaction between molecules and CSH gel, the easier the microstructure and physical properties of CSH gel are affected. To study the interface interaction between PVCap/dmapma and CSH gel more accurately, the interface interaction between its monomer molecules and CSH gel is first studied. The interaction and adsorption of molecules with CSH gel can be described in more detail by studying the movement of functional groups in capillary pores. Then, the movement of functional groups of dmapma and N-vcap in capillary pores and their effects on the microstructure of CSH are studied.

#### The movement of functional groups in dmapma and its influence on the microstructure of CSH gel

The system is divided into 200 intervals along the Z-axis by the “fix ave/chunk” command, and the distribution probability of specific atomic groups in each interval is calculated. Then, the average distribution probability of specific atomic groups along the Z-axis within 2000 ps is calculated. By counting the average distribution probability of functional groups, we can qualitatively analyze the movement state of functional groups in the capillary pores.

**Figure 6:**
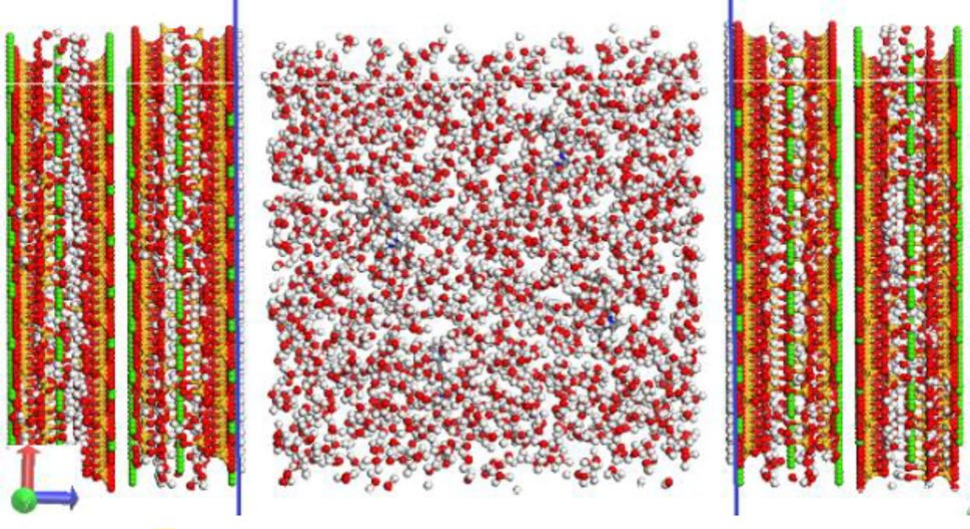
Reference lines of solid–liquid interface.

To model the real pores in CSH gel, the silicon-oxygen tetrahedron at the solid–liquid interface is hydroxylated, and the hydroxyl is perpendicular to the XY plane. The midpoints of the outermost hydroxyl groups are defined as the reference point of the solid–liquid interface, and the line connecting all the midpoints is defined as the reference line of the solid–liquid interface. According to the measurement of VMD (Visual Molecular Dynamics) software, the solid–liquid interface reference lines on the left and right sides of the initial model are located at 3.00 nm and 6.30 nm in the Z-axis (as shown in Fig. 6). During the simulations, when the CSH gel is released from fixation, the reference lines will move towards the solution area because of the coupling between solid and liquid phases.

##### Study on the distribution of different functional groups in capillary pores

**Figure 7:**
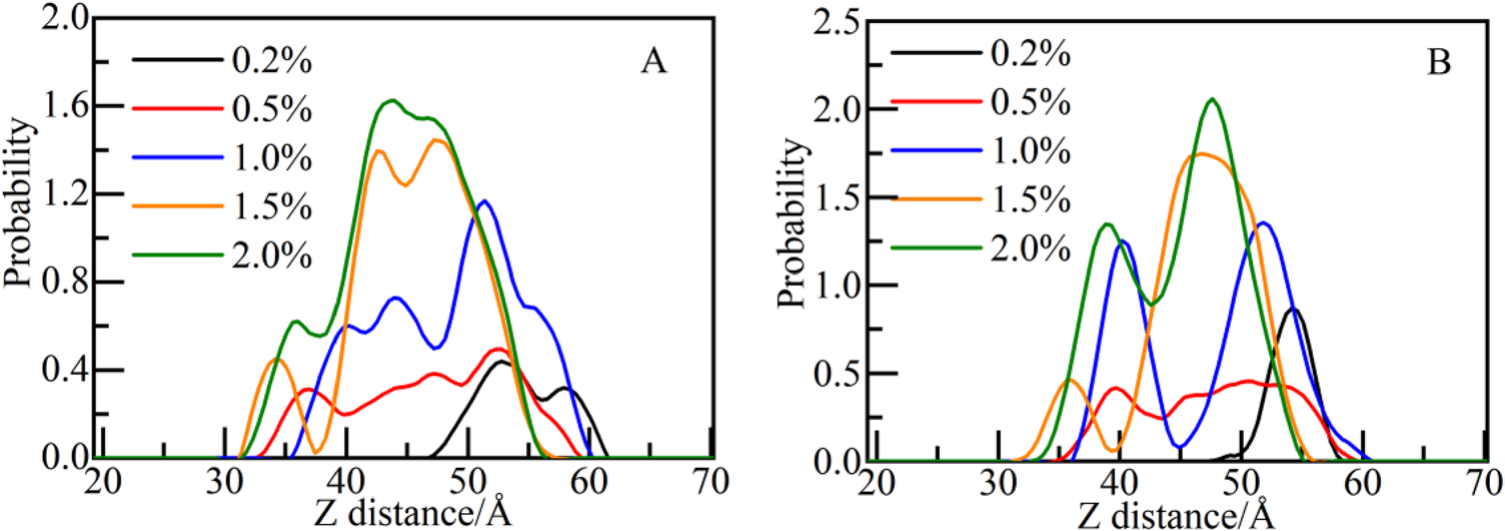
Distribution probability of functional groups along the Z-axis: (**A**) Choline group and (**B**) amide group.

Dmapma only contains two functional groups of choline and amide. The distribution probability of choline group and amide group in capillary pore with dmapma concentrations from 0.2 to 2.0% is calculated (as shown in Fig. 7). At 0.2% concentration, the choline group is generally close to the solid–liquid interface on the right side. With the increase in concentration, the peak of probability curve gradually moves to the solution area (as shown in Fig. 7A). It indicates that when dmapma concentration is low, the choline group tends to be distributed near the surface of CSH gel. Compared with the choline group, the probability peak of amide group at 0.2% concentration is higher and closer to the right side interface (as shown in Fig. 7B). It indicates that amide group has a more obvious tendency to aggregate on the surface of CSH gel at low concentrations. With the increase in concentration, amide group also shows a trend of aggregation in the solution area.

Therefore, it can be inferred that at low concentrations of dmapma, strong hydrogen bond attraction between choline and amide groups and the surface of CSH gel is form, and obvious interaction with CSH gel can be observed.

##### Effect on the microstructure of CSH gel and spatial correlation analysis between functional groups and calcium ions

**Figure 8:**
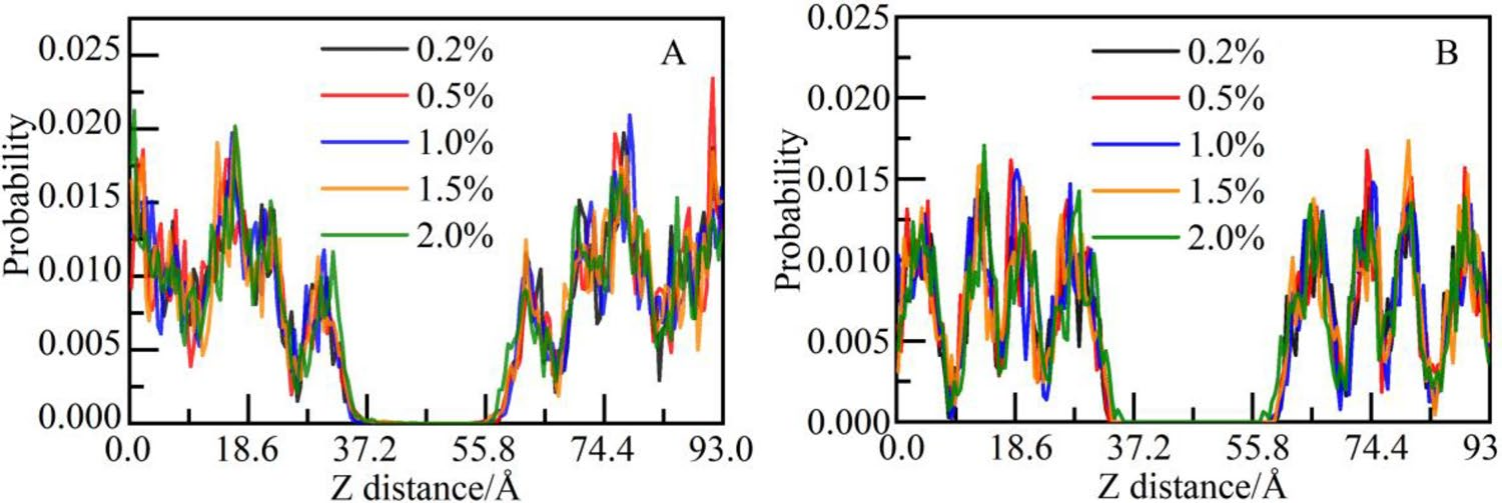
Distribution probability of CSH constituent atoms along the Z-axis: (**A**) Ca and (**B**) Si.

The distribution probability of calcium and silicon atoms in CSH gel along the Z-axis is calculated, as shown in Fig. 8. Although dmapma with different concentrations has different movement states, it has little influence on the distribution of calcium ions (as shown in Fig. 8A). Only at the concentration of 2.0%, do calcium ions have a slight tendency to move toward the solution area. The distribution of silicon atoms is unaffected basically (as shown in Fig. 8B).

The radial distribution function (RDF) describes the probability of the occurrence of other atoms at a distance of *r*(*Å*) from the central atom, which can effectively characterize the degree of ordering and microstructure characteristics of crystal. RDF is used to analyze the spatial correlation between functional groups and calcium ions as well as the aggregation degree of molecules. The equation is shown as (1)^47^:

1 $$g(r) = \frac{dN}{{4\rho \pi r^{2} dr}}$$

where, *g*(*r*) is the probability of the particular atoms occurring at a distance *r* from the central atom, dimensionless; *dN* is the number of particular atoms in the range *r* to *r* + *dr* from the center atom; $\rho$ is the density of particular atom; ${4\pi r}^{2}dr$ is the volume of the spherical layer of space around the central atom from *r* to *r* + *dr*.

**Figure 9:**
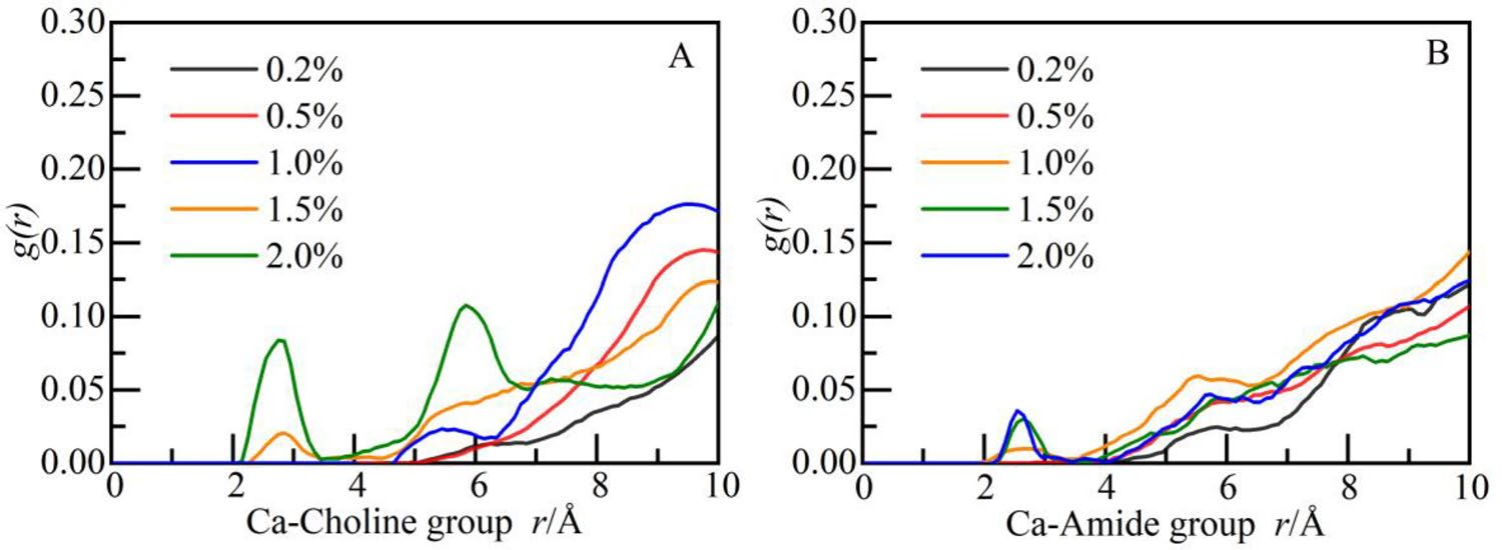
The RDF of functional groups and calcium ions: (**A**) choline group and (**B**) amide group.

To further analyze the spatial correlation between the two functional groups and calcium ions, RDF of calcium ions and choline and amide groups is calculated respectively (as shown in Fig. 9A,B). For choline group, in the low concentration (0.2–1.0%) system, there is almost no spatial correlation with calcium ions. At the concentration of 1.5%, there is a small intensity peak at 2.8 Å, and the peak increases slightly at the concentration of 2.0% (as shown in Fig. 9A). It should be noted that there are two intensity peaks at 2.8 Å and 5.8 Å at the concentration of 2.0%, indicating that there is a weak spatial correlation with calcium ions. The appearance of the second intensity peak may be due to accidental errors caused by different initial configurations of systems, but there is no impact on the analysis in this section. In section "The spatial correlation between functional groups and calcium ions", the spatial correlation between different functional groups and calcium ions will be compared and analyzed further. For amide group, when the concentration reaches 1.0%, a weak intensity peak appears at 2.8 Å (as shown in Fig. 9B). The intensity peak increases with the concentration (1.0–2.0%), but there is no strong spatial correlation with calcium ions yet. Therefore, it can be inferred that the two functional groups have no adsorption effect on calcium ions.

#### The movement of functional groups in N-vcap and its influence on the microstructure of CSH gel

**Figure 10:**
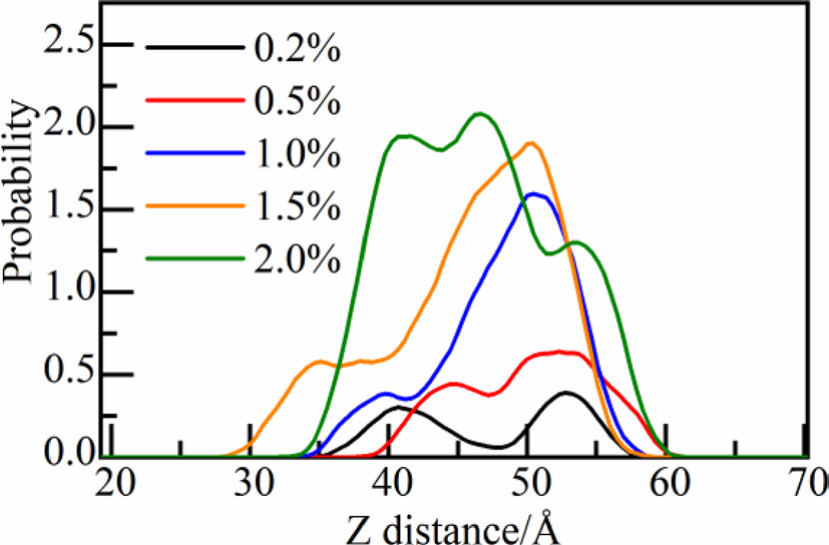
Distribution probability of caprolactam group along the Z-axis.

The distribution probability of caprolactam group along the Z-axis is calculated (only one caprolactam group exists in N-vcap), as shown in Fig. 10. Different from the choline and amide group, caprolactam group does not gather in the interface area on one side but distributes more widely in the solution area when the concentration is 0.2%. But there are still two intensity peaks near the interface on both sides. Then, with the increasing concentration of N-vcap, the intensity peak on the right side gradually increases and moves to the solution area. It indicates that the motion region of N-vcap gradually shifts from the near surface of CSH gel to the solution area.

**Figure 11:**
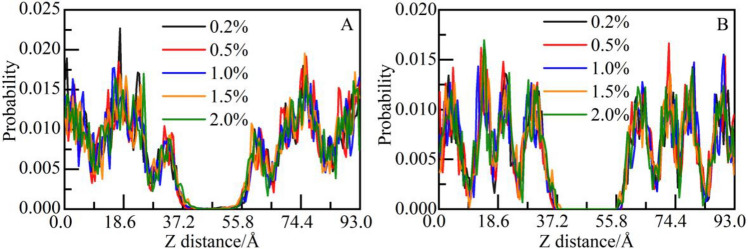
Distribution probability of CSH constituent atoms along the Z-axis: (**A**) Ca and (**B**) Si.

To further study the influence of caprolactam group on the microstructure of CSH gel, the distribution probability of calcium and silicon atoms along the Z-axis is calculated, as shown in Fig. 11. From the concentration of 0.2% to 2.0%, the distribution regularity of calcium ions is unaffected, and the intensity peaks of the five systems coincide generally (as shown in Fig. 11A). There is no phenomenon of calcium ions migration in the solution area. For silicon atoms, the intensity peaks of the five systems coincide better, indicating that the different concentrations of N-vcap do not affect the microscopic distribution of silicon chains (as shown in Fig. 11B).

**Figure 12:**
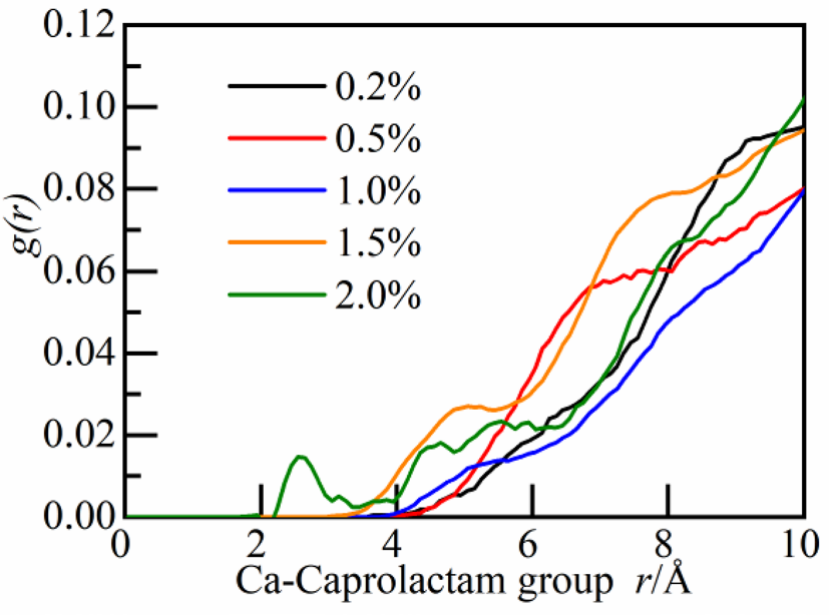
The RDF of caprolactam group and calcium ions.

To study whether calcium ions can be attracted by caprolactam group, the spatial correlation between caprolactam group and calcium ions is analyzed. As shown in Fig. 12, at the concentration of 0.2–1.5%, there is no intensity peak in the RDF curve; At the concentration of 2.0%, there is a weak intensity peak near 2.8 Å, indicating that caprolactam group has no attraction effect on calcium ions in CSH gel.

### The movement of additives in capillary pores and its influence on the microstructure of CSH gel

To study the effect of PVCap/dmapma on the microstructure of CSH gel and the decalcification of CSH gel caused by lecithin, the movement of lecithin and PVCap/dmapma at 1.5 wt% concentration in capillary pores, their influence on the microstructure of CSH gel, and the spatial correlation with calcium ions are analyzed (Previous studies pointed out that lecithin has the most negative effect on the mechanical properties of the cement slurry at the concentration of 1.5 wt%). The density of water in capillary pores is 1.0 g/cm^3^, and the model size is consistent with the model①.

#### Effect of additives on calcium ions distribution

##### Lecithin-CSH system

**Figure 13:**
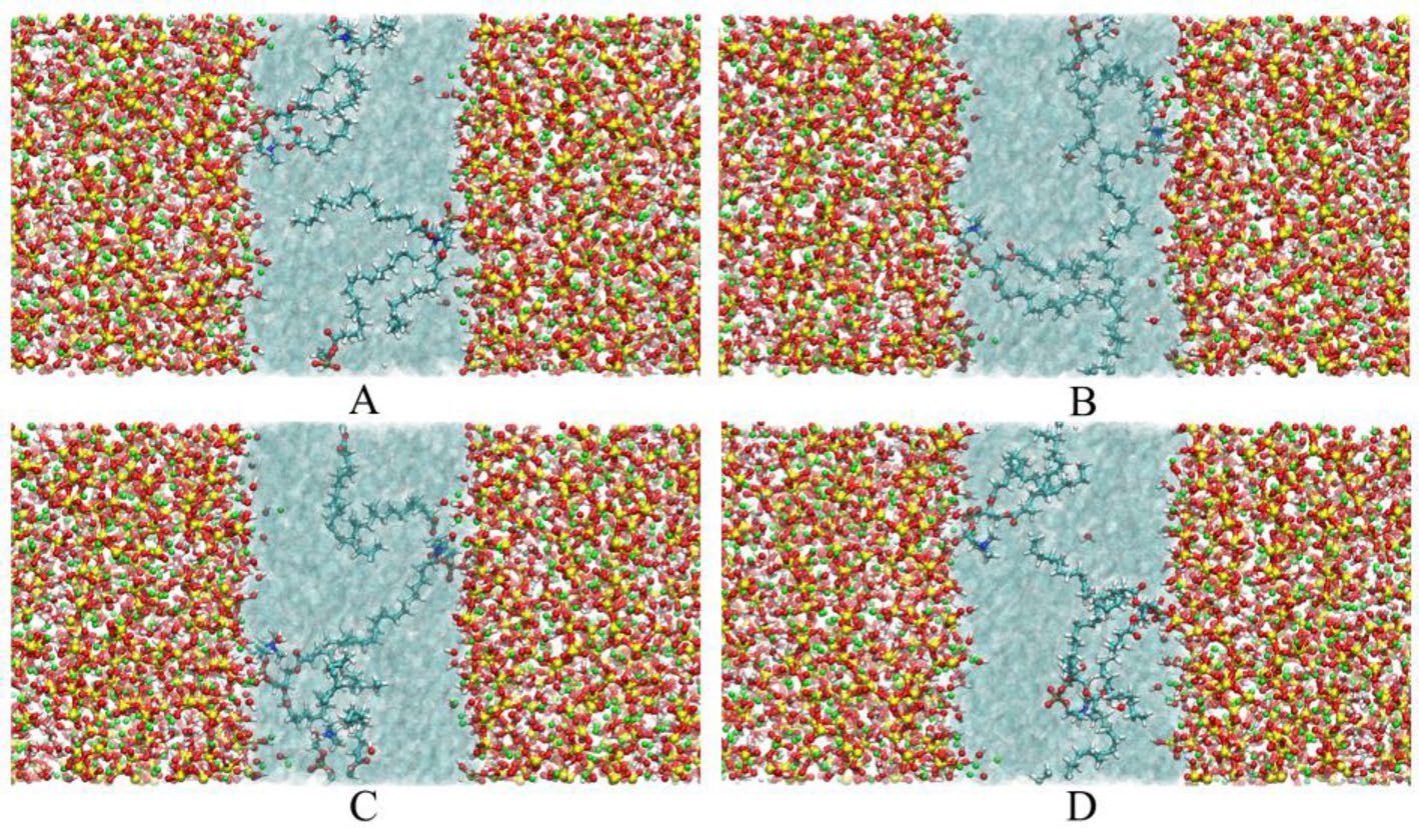
Conformation changes of Lecithin-CSH system: (**A**) 500 ps, (**B**) 1000 ps, (**C**) 1500 ps and (**D**) 2000 ps.

The conformational changes of the Lecithin-CSH system at different times within 2000 ps are shown in Fig. 13. From the whole simulation, it can be observed that two Lecithin are effectively adsorbed on the surface of CSH gel, and the other Lecithin moves freely in the solution area. Further observation shows that the phosphate groups in Lecithin are stably adsorbed on the surface of CSH gel, which led to the fixation of Lecithin. Even if the branch chains move violently in the solution area, the tight adsorption of phosphate group could not be affected.

##### PVCap/dmapma-CSH system

**Figure 14:**
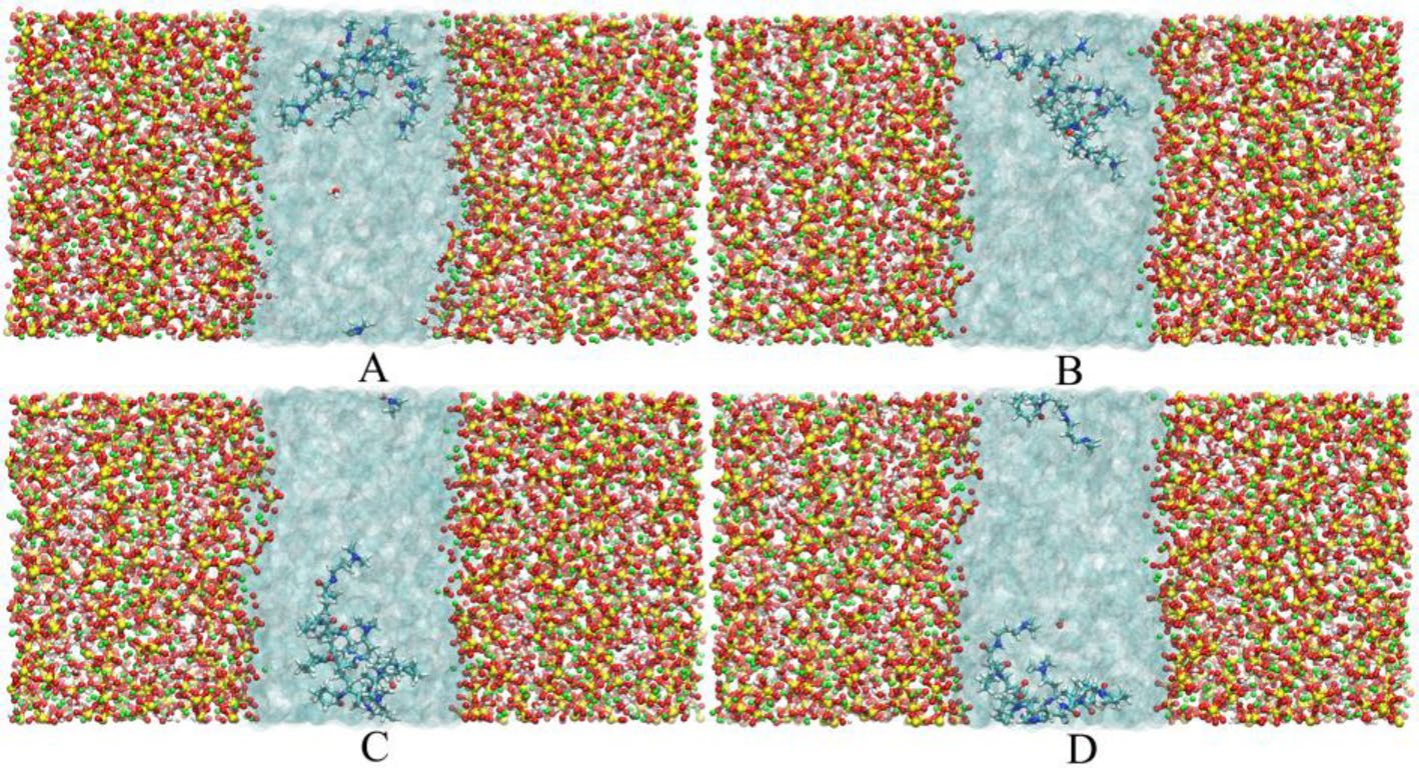
Conformational changes of PVCap/dmapma-CSH system: (**A**) 500 ps, (**B**) 1000 ps, (**C**) 1500 ps and (**D**) 2000 ps.

The conformational changes of PVCap/dmapma-CSH system at different times within 2000 ps are shown in Fig. 14. PVCap/dmapma reciprocates up and down along the Z-axis in capillary pores freely, and its branch chains are twisted constantly. Its functional groups in branch chains occasionally have an adsorption tendency on the surface of CSH gel (as shown in Fig. 14B). However, due to its weak strength and stability, PVCap/dmapma cannot establish a stable connection with CSH gel. The stronger the interaction between molecules and CSH gel, the more easily the microstructure of CSH gel will be affected. Compared with the adsorption of Lecithin on the surface of CSH gel, PVCap/dmapma is more flexible in moving and migrating in capillary pores.

**Figure 15:**
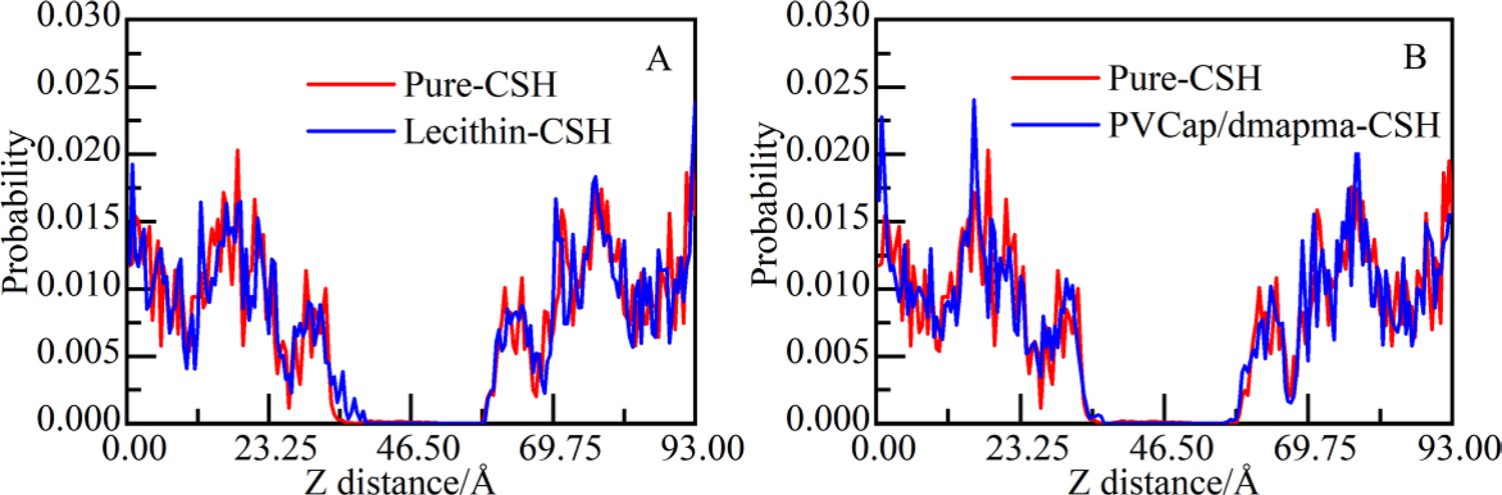
Distribution probability of calcium ions along the Z-axis: (**A**) Lecithin-CSH and (**B**) PVCap/dmapma-CSH system.

The coordination between calcium ions and oxygen atoms in silicon-oxygen tetrahedrons is the main source of maintaining the adhesion power of CSH gel. To further study the influence of the different movements of lecithin and PVCap/dmapma on calcium ions stability, the distribution probability of calcium ions along the Z-axis within 2000 ps is calculated (as shown in Fig. 15).

In the Lecithin-CSH system, it is observed that a few calcium ions near the interface migrate to the solution area (as shown in Fig. 15A). The probability peak of calcium ions near the interface decreases at different degrees compared with the Pure-CSH system. It shows that the distribution of calcium ions is affected, and the coordination between calcium ions and the silicon-oxygen tetrahedrons is weakened. The above also verifies the finding of Bu et al*.*^15^: The decalcification of CSH gel will be caused by Lecithin and thus weaken the mechanical properties of CSH gel.

For the PVCap/dmapma-CSH system, the probability peaks near the interface area are unchanged generally compared with the Pure-CSH system (as shown in Fig. 15B). There is no migration of calcium ions in the solution area. Therefore, it can be concluded that the movement of PVCap/dmapma in capillary pores will not affect the distribution of calcium ions.

#### The stability of calcium ions and CSH gel

The mean square displacement (MSD) can describe the motion intensity and diffusion trend of particles, thus characterizing the stability of system. The equation is shown as (2)^48^:

2 $$MSD = \frac{1}{N} \, \sum\nolimits_{i}^{N} {|R_{i} (t) - R_{i} (0)|^{2} }$$

where, *MSD* is the mean square displacement of atoms; *R*_*i*_(0) is the initial position of the atoms; *R*_*i*_(*t*) is the position of the atoms at time* t*; *N* is the total number of the atoms.

**Figure 16:**
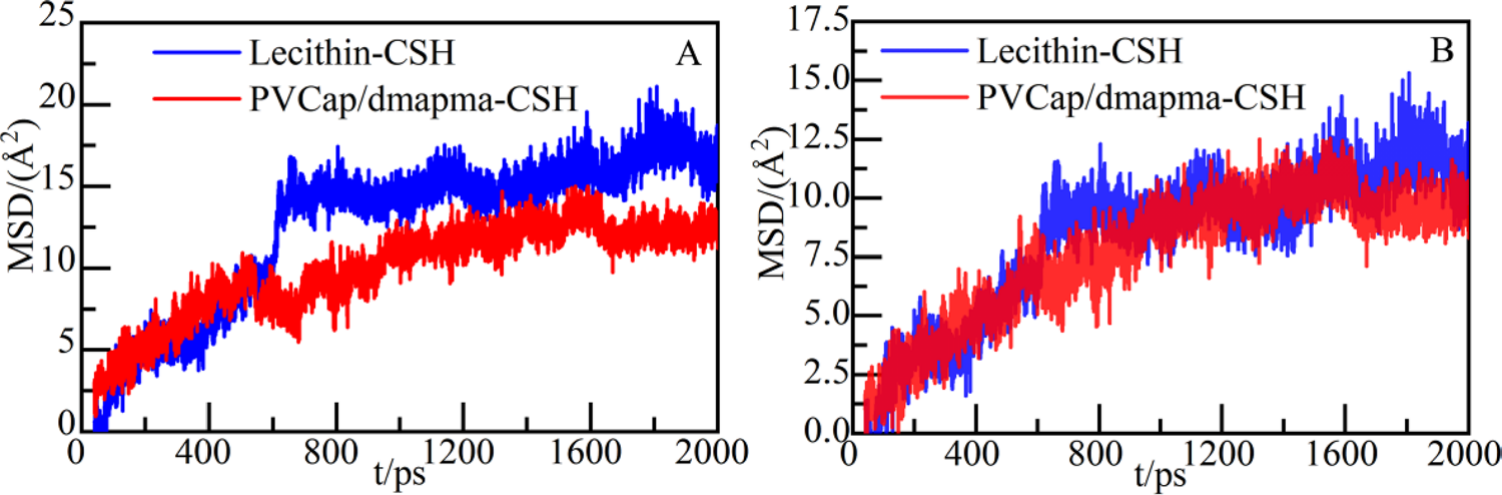
MSD: (**A**) calcium ions and (**B**) CSH gels.

To further analyze the stability of calcium ions, the MSD of calcium ions in the two systems is calculated (as shown in Fig. 16A). Compared with the PVCap/dmapma-CSH system, the MSD of calcium ions in the Lecithin-CSH system increases more sharply, reaching about 17Å^2^ at 2000 ps. It shows that the calcium ions in the Lecithin-CSH system have a stronger diffusion tendency and instability in dynamics.

Then, the MSD of the overall structure of CSH gel is counted to analyze the overall stability of CSH gel. As shown in Fig. 16B, compared with PVCap/dmapma-CSH system, the MSD of CSH gel in the Lecithin-CSH system has a wide change range and a higher overall value. It indicates that the stability of CSH gel in this system is poor in terms of dynamic performance. The MSD of CSH gel in the PVCap/dmapma-CSH system shows a steady upward trend, and even declines and stabilizes gradually after 1600 ps. It shows that the overall structure of CSH gel in the PVCap/dmapma-CSH system is more stable than the Lecithin-CSH system.

#### The spatial correlation between functional groups and calcium ions

To explore the reasons why calcium ions show different dynamic characteristics, the spatial correlation among all of the functional groups in the two molecules and calcium ions is studied in this section. Virtual centroids of phosphate group in Lecithin, amide group and choline group in PVCap/dmapma are defined, and RDF between calcium ions and the centroids of functional groups is calculated.

**Figure 17:**
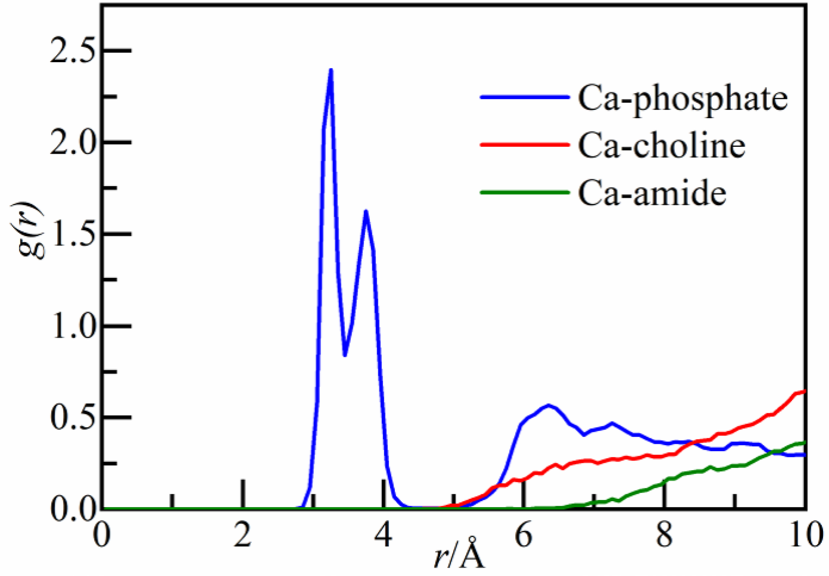
RDF of functional groups and calcium ions.

As shown in Fig. 17, the RDF of phosphate group and calcium ions shows obvious intensity peaks near 3.2 Å and 3.8 Å. It indicates a strong spatial correlation and attraction effect between phosphate group and calcium ions, which reflects the chelating effect at the macro level. Combined with Figs. 15A and 16A, It can be inferred that phosphate group is the main reason for the migration and instability of calcium ions in CSH gel. The RDF of amide and choline group and calcium ions does not show obvious intensity peaks, indicating that there is almost no spatial correlation between the two functional groups and calcium ions. According to the analysis in Section "Effect of additives on calcium ions distribution" and "The stability of calcium ions and CSH gel", when PVCap/dmapma moves in capillary pores of CSH gel, the choline group and amide group will not cause the decalcification of CSH gel and have little impact on the stability of calcium ions.

In previous research^15^, experimental and simulation findings suggested that a rise in lecithin incorporation in the cement slurry will lead to worse decalcification of cement, thus more damage to the 28-day long-term compressive strength of cement. On the microscopic scale, a stronger spatial correlation between admixtures and calcium ions means a stronger adsorption effect of admixtures on calcium ions, which makes calcium ions unstable and easier to migrate toward the gel surface, thus causing more serious decalcification. Based on the performance of PVcap/dmapma, it is predicted that PVcap/dmapma will not harm the mechanical properties of cement compared to lecithin. Detailed research will be conducted in Section "Effect of PVCap/dmapma on mechanical properties of CSH gel".

According to the previous studies^15^, the chelation of phosphate group and calcium ions will prevent the formation of calcium salt crystals. It may be the main source of the retarding effect of Lecithin. Then, Lecithin also may be adsorbed on the surface of cement particles, thus hindering the hydration process. There is no spatial correlation between choline and amide groups and calcium ions under the molecular simulation perspective, and they will not chelate calcium ions or lead to the decalcification of CSH gel. Therefore, it is inferred that PVCap/dmapma has a weak retarding effect on cement slurry, and the probability of adsorption on the surface of cement particles hindering the hydration process is low.

Organic phosphate is often used as cement retarders^49^, and its retarding effect is 4 to 7 times that of inorganic phosphate. Organic phosphate exhibits a potent chelating capability towards calcium and aluminum ions, which can significantly delay the coagulation of cement. In European and American countries, if the cement slurry mixed cannot be used up completely every Friday, the remaining cement slurry will be discarded directly due to the rest day in the past. However, in the environmental protection laws nowadays, such behavior is prohibited strictly. Therefore, workers can terminate the hydration of cement completely by adding a small amount of organic phosphate into the cement slurry. The cement slurry can still maintain fluidity after the weekend, at this time, it can be mixed with the cement slurry mixed freshly in proportion, which can still be used for conventional construction^50^. In 1989, the United States Monsanto Company introduced an organic phosphate retarder, and the hydration of cement is even terminated completely within a few weeks^51^. The above research shows a strong spatial correlation between phosphate group in Lecithin and calcium ions from the perspective of molecular simulation, which just reflects the strong chelation of organic phosphate on calcium ions at the macro level. Therefore, it is inferred that the role of Lecithin in cement slurry may be similar to that of organophosphate retarder.

The previous studies^15^ tested the mechanical properties of CSH gel containing 1.5 wt% Lecithin after 28 days of low-temperature curing. They found that its compressive strength and elastic modulus are significantly lower than those of CSH gel without Lecithin. And they believed that the addition of Lecithin will lead to the decalcification of CSH gel, which is the main reason for this result. Based on the above analysis, we believe that there are two reasons why Lecithin affects the mechanical properties of CSH gel: ① The migration of calcium ions in CSH gel will be caused by Lecithin (decalcification of CSH gel); ② The super retarding effect of Lecithin causes CSH gel that can be hydrated completely in 28 days cannot be fully hydrated, which affects the long-term mechanical properties of cement. Therefore, this is also why Lecithin is unsuitable for hydrate formation cementing.

### Effect of PVCap/dmapma on mechanical properties of CSH gel

The effect of PVCap/dmapma on the mechanical properties of CSH gel is studied in this section: First, the possibility of PVCap/dmapma stable existence in CSH gel and its effect on the atomic structure evolution of CSH gel are analyzed. Then, use the “deform” command to stretch and compress the CSH gel along X, Y, and Z-axes at the tensile and compressive strain rates of 0.008/ps respectively (timesteps 0.1 fs).

#### Intercalation possibility of PVCap/dmapma in CSH gel

First, calculate the binding energy between PVCap/dmapma and CSH gel, and analyze the adsorption possibility of PVCap/dmapma in CSH gel from the thermodynamic view. The Equations are shown in (3) and (4)^52^:

3 $$E_{{{\text{interaction}} }} = E_{{{\text{total}} }} - (E_{{{\text{CSH}}}} + E_{{{\text{admixtures}}}} )$$

4 $$E_{{{\text{binding}}}} = - E_{{{\text{interaction}} }}$$

where, *E*_interaction_ is the interaction energy of the additive-CSH system in equilibrium, kJ/mol; *E*_total_ is the total energy of the additive-CSH system in equilibrium, kJ/mol; *E*_CSH_ is the energy of CSH gel without additive in equilibrium, kJ/mol; *E*_admixtures_ is the energy of the additive in equilibrium, kJ/mol; *E*_binding_ is the binding energy, and a positive value indicates adsorption, kJ/mol.

As shown in Table 1, the binding energy between PVCap/dmapma and CSH is positive, which indicates that there is a possibility of PVCap/dmapma adsorption in CSH gel or silicon chain defects from the thermodynamic view.

**Table 1:** Calculation results of the binding energy.

	*E*_total_	*E*_admixtures_	*E*_CSH_	*E*_binding_
Energy/(kJ/mol)	− 401,923.745	3824.439	− 405,730.834	17.35

#### Compression constitutive relation of CSH gel

CSH gel has an amorphous structure in the real state, and its structure shows irregular characteristics in all directions. At present, the CSH models constructed by scholars are not perfect enough, so it does not have credibility to conduct mechanical performance tests only in a single direction. To evaluate the mechanical properties of CSH gel model more accurately, it is necessary to conduct compression and tensile tests in all directions.

The CSH gel model shows anisotropy in each direction: in the X and Y directions, the bond strength is mainly provided by the connection of silicon chain, and the destruction of Si–O bond requires strong stress and energy excitation^53,54^. In the Z direction, it mainly depends on the calcium ion layer and the oxygen in silicon chain to form the calcium-oxygen octahedral coordination structure to maintain the bonding force,which is more vulnerable to destruction. The specific performance is that the compressive strength and Young's modulus are lower than the X and Y directions.

**Figure 18:**
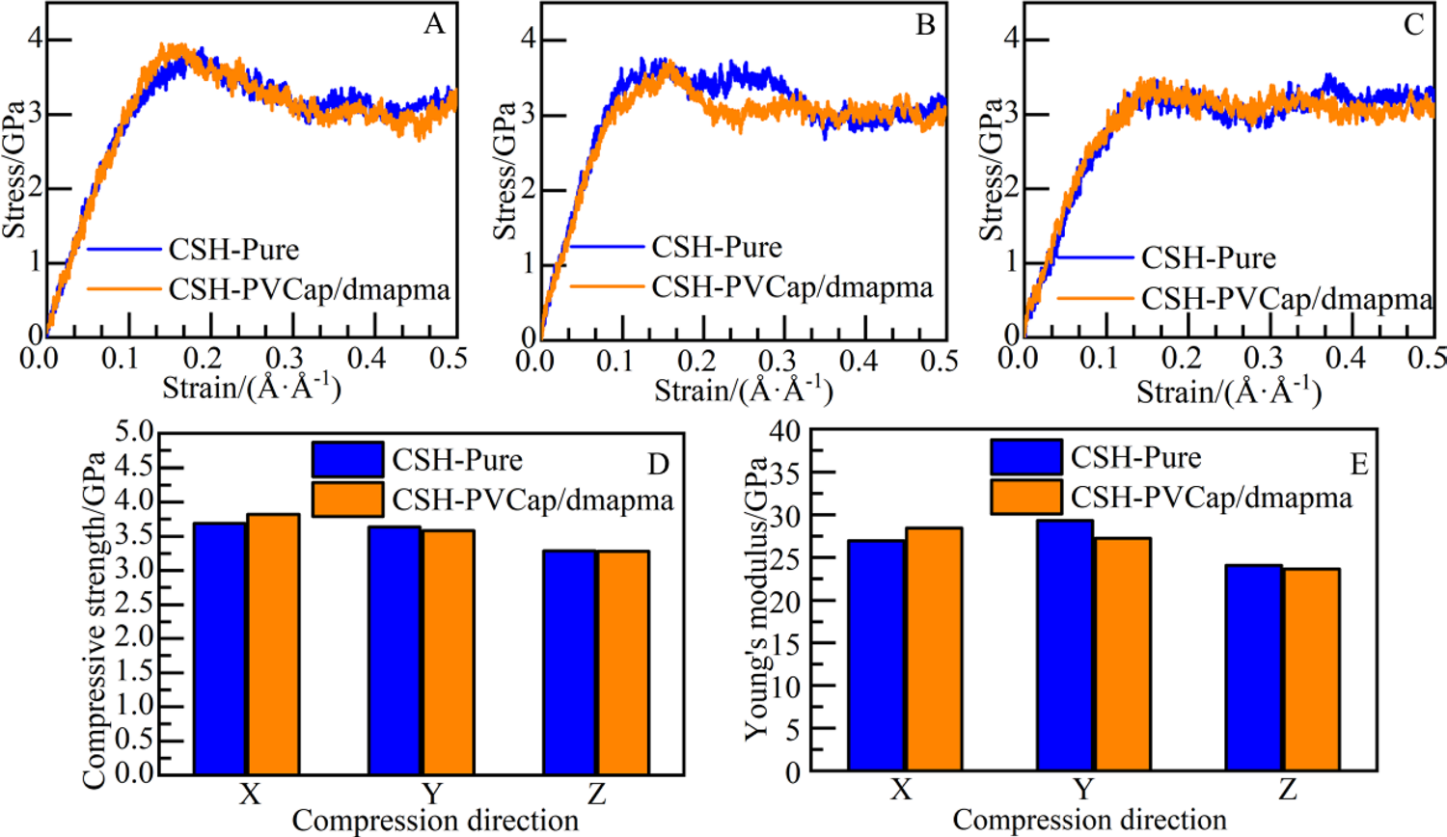
Compressive mechanical properties: stress–strain curve: (**A**) X-axis, (**B**) Y-axis, and (**C**) Z-axis. (**D**) Compressive strength, (**E**) Young's modulus.

Compress CSH gel in X, Y, and Z directions, and output the compression stress–strain curves of the CSH-PVCap/dmapma and CSH-pure systems, as shown in Fig. 18A–C. It should be noted that the principle of the “deform” command is to compress the atoms by changing the volume of the model. The height of the compressed direction becomes lower, and the length of the other two directions becomes longer. After the compression failure of CSH, more and more atoms continue to pile up under stress. So, the compressive stress will not significantly decline after compression failure.

During the compression process, the mechanical responses of stress–strain curves in all directions of the two systems at the elastic stage coincide generally. The compressive strength and Young's modulus (the slope of the elastic stage) are calculated by fitting on the elastic stage of stress–strain curves, as shown in Fig. 18D,E. The constitutive relation of the CSH-PVCap/dmapma and CSH-pure systems at the elastic stage is fitted by the geometric average of stress–strain curves in all directions, as shown in Eqs. (5) and (6):

5 $$\sigma = 26.07*\varepsilon (0 < \varepsilon < 0.120)$$

6 $$\sigma = 26.39*\varepsilon (0 < \varepsilon < 0.121)$$

As shown in Fig. 18D, for the two systems, X-axis compressive strength is slightly stronger than Y-axis compressive strength, and Z-axis compressive strength is the weakest. This also verifies the above analysis: breaking the connection of silicon chains in the X and Y-axes requires stronger stress. As shown in Fig. 18D, the X-axis compressive strength of the CSH-PVCap/dmapma system is slightly stronger than that of the CSH-pure system. Y-axis and Z-axis compressive strength are generally consistent with that of the CSH-pure system.

As shown in Fig. 18E, X-axis Young's modulus of the CSH-pure system is 26.97GPa, and that of the CSH-PVCap/dmapma system is 28.46GPa; Y-axis Young's modulus of the CSH-pure system is 29.34GPa, and that of the CSH-PVCap/dmapma system is 27.26GPa; Young's modulus is not affected in the Z-axis. This indicates that the existence of PVCap/dmapma slightly increases X-axis Young's modulus, but slightly decreases Y-axis Young's modulus. In general, PVCap/dmapma has little effect on the compressive mechanical properties of CSH gel. In the two systems, the compressive strength and Young's modulus show a positive correlation: take the CSH-PVCap/dmapma system as an example, take the compressive strength *P*_*c*_ as the independent variable, and Young's modulus *E* as the dependent variable, the fitting relation is shown in Eq. (7):

7 $$E = \, 8.9756*P_{c} - 5.5012\left( {{\text{R}}^{{2}} { = }0.9554} \right)$$

#### Tensile constitutive relation of CSH gel

**Figure 19:**
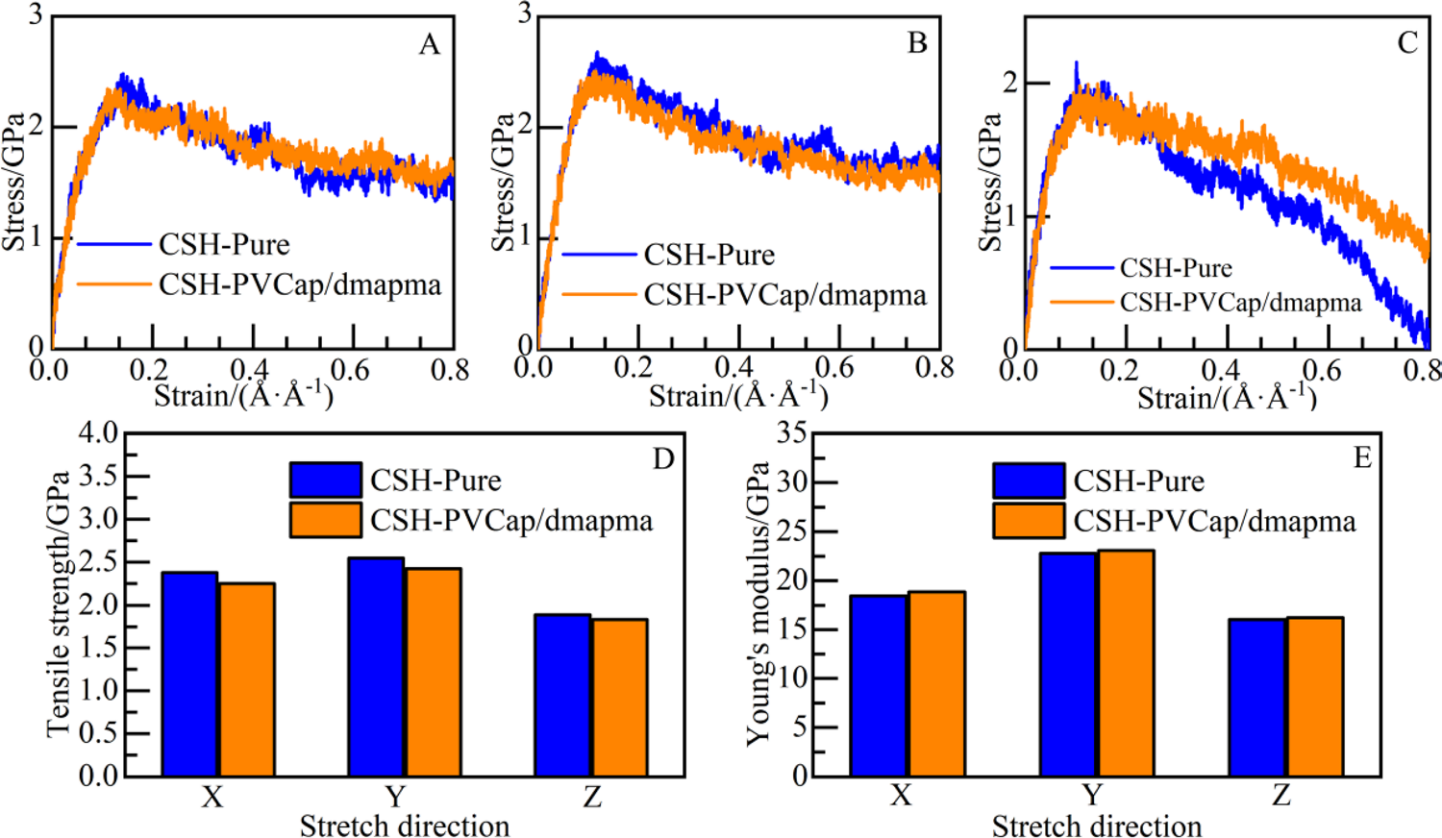
Tensile mechanical properties: stress–strain curve: (**A**) X-axis, (**B**) Y-axis, and (**C)** Z-axis. (**D**) Tensile strength, (**E**) Young's modulus.

Stretch CSH gel in X, Y, and Z directions, and output the tensile stress–strain curves of the CSH-PVCap/dmapma and CSH-pure systems (as shown in Fig. 19A–C). The mechanical responses of the two systems at the elastic stage in the X, Y, and Z-axes coincide generally. However, at the post-yield stage in the Z-axis, after the CSH-PVCap/dmapma system reaches the yield point, the rate of stress reduction slows down (as shown in Fig. 19C). It indicates that the incorporation of PVCap/dmapma will delay the fracture process of CSH gel.

The tensile strength and Young's modulus in each direction are calculated by fitting the elastic stage of stress–strain curves. The constitutive relation of the CSH-pure and CSH-PVCap/dmapma systems at the elastic stage is as shown in Eqs. (8) and (9):

8 $$\sigma = 18.87*\varepsilon (0 < \varepsilon < 0.122)$$

9 $$\sigma = 19.17*\varepsilon (0 < \varepsilon < 0.121)$$

As shown in Fig. 19D, for the CSH-PVCap/dmapma system, the tensile strength in the X and Y-axes is slightly weaker than the CSH-pure system, and the Z-axis generally coincides with the CSH-pure system. As shown in Fig. 19E, Young's modulus of the CSH-PVCap/dmapma system in the X, Y, and Z-axes has a small increase compared with the CSH-pure system. So, it can be considered that PVCap/dmapma has no effect on Young's modulus of CSH gel. In the two systems, the tensile strength and Young's modulus present a positive correlation: take the CSH-PVCap/dmapma system as an example, take the tensile strength *P*_*a*_ as the independent variable, and Young's modulus *E* as the dependent variable, the fitting relation is shown in Eq. (10):

10 $$E = 10.652*P_{a} - 3.7362(R^{2} = 0.869)$$

**Figure 20:**
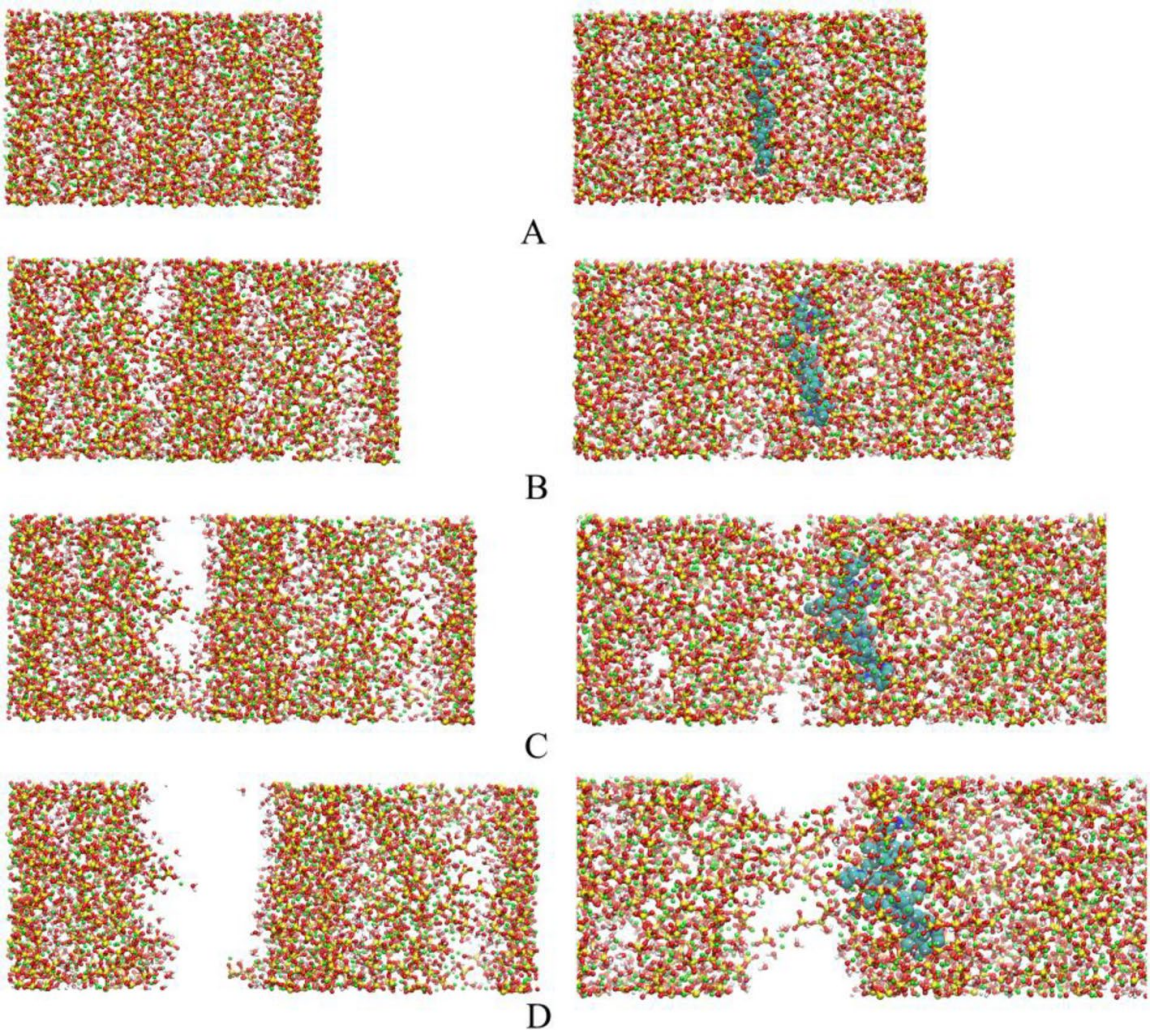
Atomic structure evolution of CSH gel in Z-axis tensile: (**A**) 0.2 Å/Å, (**B**) 0.4 Å/Å, (**C**) 0.6 Å/Å, and (**D**) 0.8 Å/Å. (Left is CSH-pure, Right is CSH-PVCap/dmapma).

To explore the reason why PVCap/dmapma delays the fracture failure of CSH gel in the Z-axis, the atomic structure evolution of CSH gel during the tensile failure is shown in Fig. 20. When the tensile strain is between 0.2 Å/Å and 0.4 Å/Å, there is no obvious difference in the structure of CSH gel in the two systems (as shown in Fig. 20A,B). Most of the fracture defects have been found in the interlayer region of the CSH-pure system when the tensile strain reaches 0.6 Å/Å. But most silicon chains are still connected at the fracture of the CSH-PVCap/dmapma system (as shown in Fig. 20C). the CSH-pure system has completely fractured when the tensile strain reaches 0.8 Å/Å, but some silicon chains are still connected in the CSH-PVCap/dmapma system (as shown in Fig. 20D). Therefore, it can be concluded that the existence of PVCap/dmapma enhances the bonding mechanism between the silicon chains at the fracture and delays the tensile failure in the interlayer region. Eftekhari^55^ also got a similar conclusion in the study of tensile failure of the CSH and carbon nanotubes composite systems: the bridging effect of carbon nanotubes in the interlayer silicon chain significantly improves the mechanical properties and ductility of the whole system. Therefore, it is speculated that PVCap/dmapma may enhance the toughness and ductility of CSH gel.

In general, the existence of PVCap/dmapma in CSH gel will not cause obvious changes in its microstructure. In the tensile or compression failure process, only the Z-axis tensile process shows stronger ductility, and the constitutive relations in other directions have no difference generally. The above shows that CSH gel is not sensitive to PVCap/dmapma. PVCap/dmapma will not greatly impact the microstructure and mechanical properties of CSH gel.

## Conclusions

Through simulation research and theoretical analysis, the following conclusions are obtained:

1. Based on the ^29^Si-NMR spectrum, thermogravimetric analysis, the low-temperature cement slurry system formula, and the combination with GCMC simulation, we established a CSH gel model that is closer to the hydration product of the actual deepwater oilwell cement, providing a good reference for the further application of molecular simulation technology in the field of oil and gas cementing.
2. When Lecithin moves in the capillary pores, due to the existence of phosphate group, the whole molecule is firmly adsorbed on the surface of CSH gel. There is a strong spatial correlation between phosphate group and calcium ions, which causes the decalcification of CSH gel. The role of Lecithin in cement slurry may be similar to that of organophosphate retarders. There are two reasons why Lecithin affects the mechanical properties of CSH gel: ① The migration of calcium ions in CSH gel will be caused by Lecithin (that is, decalcification); ② The super retarding effect of Lecithin causes CSH gel that can be hydrated completely in 28 days cannot be fully hydrated, which affects the long-term mechanical properties of cement.
3. PVCap/dmapma shows strong dynamic behavior within capillary pores, exhibiting flexibility and mobility, while failing to establish an effective interfacial connection with the CSH gel. The choline and amide groups of PVCap/dmapma exert little influence on the stability of calcium ions, lacking any spatial correlation with calcium ions. They will not chelate calcium ions or lead to the decalcification of CSH gel. Therefore, it is inferred that PVCap/dmapma has a little probability of adsorption on the surface of cement particles to hinder hydration process.
4. PVCap/dmapma has the possibility of intercalation in CSH gel, and will not cause significant changes in its microstructure. PVCap/dmapma has little effect on the compressive strength and Young's modulus of CSH gel, but it is possible to improve the toughness and ductility of CSH gel in tensile properties. CSH gel is not sensitive to PVCap/dmapma, thus PVCap/dmapma has the employ potential as a hydrate dissociation inhibitor for cement slurry system.

## Data Availability

The datasets used and analysed during the current study available from the corresponding author on reasonable request.
